# Promising effects of treatment with flotation-REST (restricted environmental stimulation technique) as an intervention for generalized anxiety disorder (GAD): a randomized controlled pilot trial

**DOI:** 10.1186/s12906-016-1089-x

**Published:** 2016-03-25

**Authors:** Kristoffer Jonsson, Anette Kjellgren

**Affiliations:** Department of Psychology, Karlstad University, SE-651 88 Karlstad, Sweden

**Keywords:** Flotation-REST, Flotation-tank, Sensory isolation, Generalized anxiety disorder, Anxiety, Emotion regulation, Relaxation

## Abstract

**Background:**

During Flotation-REST a person is floating inside a quiet and dark tank, filled with heated salt saturated water. Deep relaxation and beneficial effects on e.g. stress, sleep difficulties, anxiety and depression have been documented in earlier research. Despite that treatments for generalized anxiety disorder (GAD) are effective; it is till the least successfully treated anxiety disorder, indicating that treatment protocols can be enhanced. The use of Flotation-REST as a treatment of GAD has not been researched. The aim of the present study was to conduct an initial evaluation of the effects in a self-diagnosed GAD sample.

**Methods:**

This study was a randomized, parallel group, non-blinded trial with 1:1 allocation ratio to waiting list control group (*n =* 25) or to a twelve session treatment with flotation-REST (*n =* 25). Inclusion criteria’s were: 18–65 years and GAD (as defined by self-report measures). The primary outcome was GAD-symptomatology, and secondary outcomes were depression, sleep difficulties, emotion regulation difficulties and mindfulness. Assessments were made at three time points (baseline, four weeks in treatment, post-treatment), and at six-month follow-up. The main data analyses were conducted with a two-way MANOVA and additional t-tests. Forty-six participants (treatment, *n =* 24; control, *n =* 22) were included in the analyses.

**Results:**

A significant *Time x Group* interaction effect for GAD-symptomatology [*F*_(2,88)_ = 2.93, *p* < .001, *η*_*p*_^*2*^ = .062] was found. Further analyses showed that the GAD-symptomatology was significantly reduced for the treatment group (*t*_*(*23)_ = 4.47, *p* < .001), but not for the waiting list control group (t_(21)_ = 0.98, *p* > .05), when comparing baseline to post-treatment scoring. Regarding clinical significant change, 37 % in the treatment group reached full remission at post-treatment. Significant beneficial effects were also found for sleep difficulties, difficulties in emotional regulation, and depression, while the treatment had ambiguous or non-existent effects on pathological worry and mindfulness. All improved outcome variables at post-treatment, except for depression, were maintained at 6-months follow. No negative effects were found.

**Conclusion:**

The findings suggest that the method has potential as a complementary treatment alongside existing treatment for GAD. More studies are warranted to further evaluate the treatments efficacy.

**Trial registration:**

Australian New Zealand Clinical Trial Registry: ACTRN12613001105730, Date of registration: 03/10/2013

## Background

Generalized Anxiety Disorder (GAD) is characterized by a more or less constant state of excessive worry and anxiety, with symptoms such as poor sleep, muscle tension, irritability, and fatigue [1]. GAD is a relatively common disorder, with a lifetime prevalence rating of 5.7 %, and a 12-month prevalence of 3.1 % [2]. It has been associated with considerable suffering and functional impairment, as well as high costs for society [3]. In addition, GAD is associated with high risk of comorbidity, in which depression is the most common secondary diagnosis [4]. Several risk factors for developing GAD have been proposed, such as traumas [5], innate emotional responsiveness [6], dysfunctional interpersonal processes [7], as well as insecure attachment style [8].

Although pharmacological treatment has been shown to be effective for treating GAD [(e. g.) [9]], psychotherapies are usually preferred over medication by patients and clinicians alike [3]. Several psychotherapeutic interventions for GAD have been developed and most of them are based on cognitive behavioral therapy (CBT) [10]. Many of the contemporary CBT conceptualizations of GAD propose that the core problem in the disorder is worry, which serves as an avoidance strategy of internal experiences, while others emphasize difficulties in emotion regulation as the central problem [11]. In addition, low levels of trait mindfulness have been proposed to play an important role in the psychopathology of GAD [12]. This is supported by clinical studies where mindfulness training has proven to be a robust intervention to reduce anxiety symptoms [13] as well as by being applied successfully as a primary treatment for GAD [14].

Although CBT is to be considered an effective method for treating GAD, it is not as effective as for other types of anxiety disorders [15]. It has also been pointed out that the majority of treated GAD patients only reach partial remission, which further highlights that many of those which respond to treatment still have residual symptoms [16]. Taken together this suggest that treatment protocols might be improved, by for example adding some form of complementary treatment. In addition, it could be valuable to evaluate alternative forms of treatment for GAD, since some patients prefer complementary and alternative medicine (CAM) to conventional interventions or use it alongside first-line treatments [17].

The present study evaluates a CAM therapy, namely flotation-REST (Restricted Environmental Stimulation Technique) as an intervention for a self-diagnosed GAD-sample. Flotation-REST has to date not been studied primarily as an intervention for GAD, but the method has in earlier research been shown to reduce stress [18], anxiety [(e. g.) [19]] and depression [(e. g.) [20, 21]], as well as to alleviate many of the symptoms associated with GAD, such as sleep difficulties [22, 23], fatigue [(e. g.) [20], and muscle tension pains [(e. g.) [24]]. During flotation-REST a person is lying horizontally, face up, inside a quiet and dark tank, filled with salt (magnesium sulphate) saturated water held at 35 °C (95 F). The water has high buoyancy, which makes it possible to float comfortably on the back inside of the tank. It has been suggested that the method achieves its beneficial effects through deep relaxation that is induced by sensory isolation, and effects of treatment are manifested without much effort or specific instructions [25]. Contemporary research on flotation-REST has established a treatment protocol consisting of 12 sessions (á 45 min) twice a week, which has been suggested to be sufficient to reach the desired therapeutic effects [21]. Flotation-REST has also been used successfully as a treatment for chronic pain conditions [24, 26], burn-out syndrome [20, 23], as well as a preventive health-care intervention [19]. There are also pilot studies showing promising results when flotation-REST is combined with psychotherapy [27, 28]. Taken together earlier research indicate that flotation-REST might have potential as an complementary or alternative intervention for GAD, but due to the focus and methodological shortcomings in earlier studies further research need to establish the effectiveness of flotation-REST as treatment for GAD. As a first step to clarify this the present study aims to conduct an initial evaluation to gather information which could guide further research in the field, and answer the question if it could be fruitful to study flotation-REST as a treatment for GAD. The main aim of the study was to evaluate a 12 session treatment program of flotation-REST on suggested core problems (pathological worry; emotional regulation difficulties; and low mindfulness), the most common secondary diagnosis (depression), as well as the symptomatology (e. g. fatigue, sleep-difficulties, muscle tension) associated with GAD. The primary outcome in this study was the general level of GAD-symptomatology, and the secondary outcomes were depression, sleep difficulties, emotion regulation difficulties and mindfulness. In addition, deviation from normal state during the flotation session was measured to assess level of subjective relaxation during the flotation sessions.

## Method

### Participants

Fifty-nine participants were recruited to the study. Recruitment was made through outpatient psychiatric care, as well as by advertisement in a local paper, stating that sufferers of prolonged anxiety problems, and with an age of 18–65 years, were welcomed to register their interest to participate in a study on relaxation in flotation tank. All recruitment was made from October 2013 to April 2014. Interested individuals who by telephone contact seemed fitting to participate (*n =* 59), were screened with the Generalized Anxiety Questionnaire 4th edition (GAD-Q-IV) and the Penn State Worry Questionnaire (PSWQ) using a cut-off score found to successfully detect generalized anxiety disorder (GAD) in both clinical and non-clinical samples [29, 30]. From the recruited sample (*n =* 59), in total fifty met the inclusion criteria’s and were randomized to either treatment condition (*n =* 25) or waiting-list control condition (*n =* 25). The randomization was conducted by the researchers, who asked the participants to take a slip of paper from a jar. The jar contained 50 paper slips numbered from 1 to 50, where odd numbers indicated an allocation to treatment condition and even numbers allocation to waiting list control condition. Allocation was performed after the baseline assessments were made. One participant in the treatment condition and three participants in the waiting list condition did not complete the study due to unknown reasons. In addition, five participants in the treatment condition did not complete the six-month follow-up assessments. Inclusion criteria’s for the present study were (a) 18–65 year of age, (b) having GAD as defined with the PSWQ (Cutoff = 45+) and/or the GAD-Q-IV (Cutoff = 5.7+). Exclusion criteria’s were (a) ongoing pregnancy, (b) having a pacemaker implant, (c) having open wounds or skin-disease, (d) epilepsy, (e) history of psychosis, (f) bipolar syndrome, (g) post-traumatic stress disorder, (h) and ongoing substance abuse (exception was made for tobacco).

**Fig. 1:**
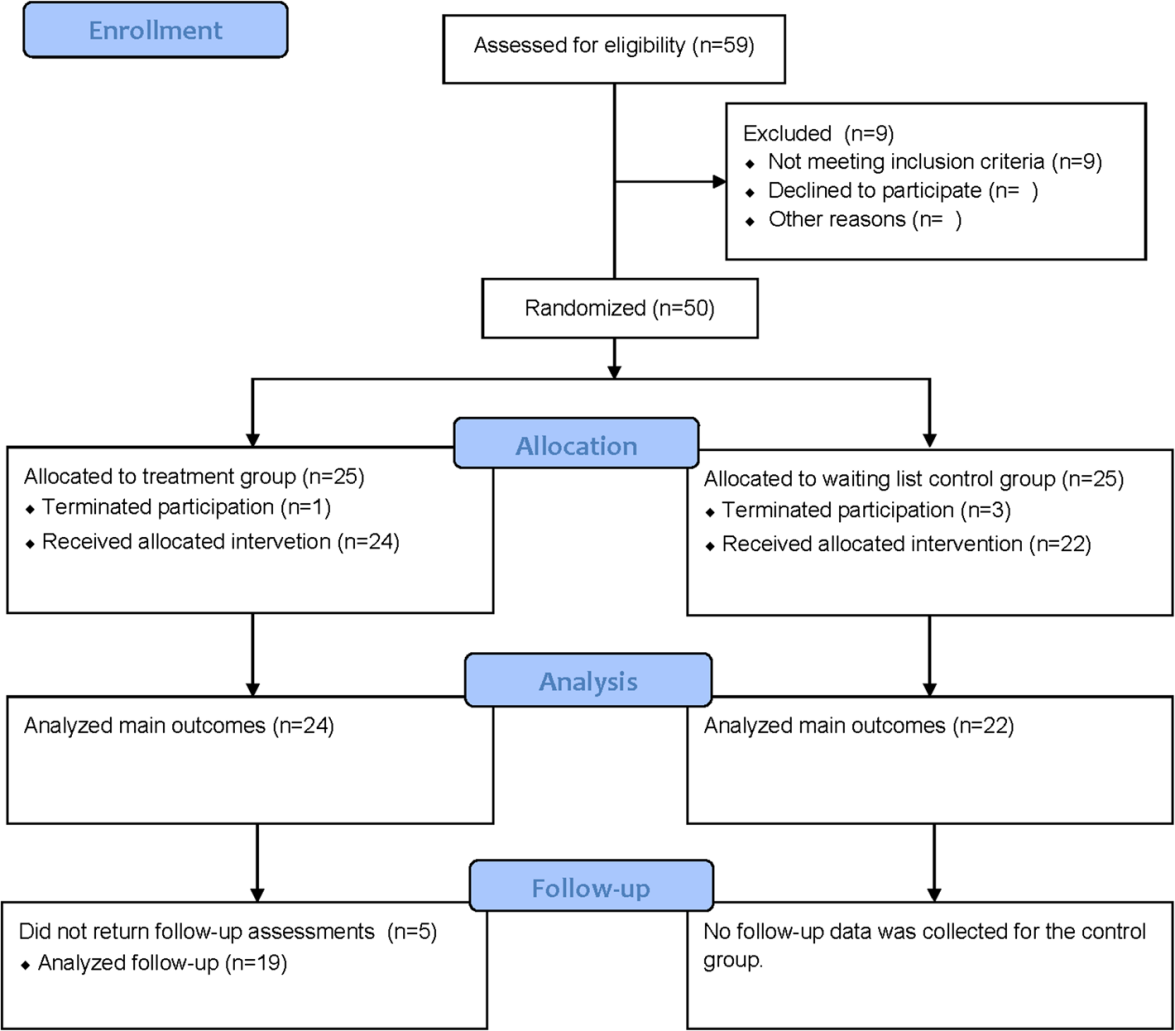
Flow diagram depicting the recruitment process. Template used for the flow diagram was downloaded from http://www.consort-statement.org/consort-statement/flow-diagram0/

Independent t-tests of the baseline assessments (See Measures) did not indicate any significant differences between the groups at baseline (*ps* > .05), except for mindfulness (MAAS), where the waiting list control group scored significantly higher than the treatment group (*p* < .05). Although the groups differed significantly on this variable, both group’s baseline scoring were low when considering normative data [31]. For means and standard deviations of the baseline scoring see Table 1. The recruitment process is depicted in Fig. 1 below.

**Table 1:** Baseline characteristics of participants included in the study

Measure	Treatment group (n = 25)	Control group (n = 25)	Total sample (n = 50)
Age M (SD)	42.71 (12.49)	43.41 (14.57)	43.04 (13.37)
PSWQ	61.88 (11.21)	57.95 (10.29)	60.00 (10.84)
GAD-Q-IV	10.01 (2.20)	9.92 (2.24)	9.87 (2.19)
MADRS-S	24.04 (7.46)	20.77 (7.35)	22.48 (7.51)
PSQI	10.58 (3.92)	9.73 (3.49)	10.17 (3.70)
DERS	101.67 (20.18)	97.64 (20.33)	99.74 (20.13)
MAAS	2.98 (0.65)	3.64 (0.79)	3.29 (0.78)
Sex	% (n)	% (n)	% (n)
Female	72 (18)	68 (17)	70 (35)
Male	28 (7)	32 (8)	30 (15)
Medication			
Yes	32 (8)	48 (12)	40 (20)
No	68 (17)	52 (13)	60 (30)
Psychotherapy			
Yes	32 (8)	36 (9)	34 (17)
No	68 (17)	64 (16)	66 (33)

Note. PSWQ = Penn State Worry Questionnaire; GAD-Q-IV = Dimensional scoring from the Generalized Anxiety Disorder Questionnaire; MADRS-S = Montgomery-Asberg Depression Rating Scale; PSQI = Pittsburgh Sleep Quality Index; DERS = Dysfunctional Emotional Regulation Scale – Total mean score; MAAS: Mindful Attention and Awareness Scale; Medication: intends regular use of anxiolytic and antidepressant drugs (yes/no); Psychotherapy: intends ongoing treatment with psychotherapist (yes/no)

### Design

This was a randomized, parallel group, non-blinded trial with 1:1 allocation ratio to waiting list control group or to a twelve session treatment with flotation-REST. The study was performed in the mid-west part of Sweden during 2013 to 2015. A two-way split plot design was performed, where *Time* with assessments baseline, 4-weeks in treatment, and after treatment, constituted the within-subject factor. *Group* (treatment; waiting list control) constituted the between-subject factor. The participants in the treatment group received a 7-week treatment program of flotation-REST that consisted of in total 12 treatment sessions (á 45 minutes). The first treatment session was administered January 2014 and the last July 2014. A test battery of validated self-report measures (See Measures) was administered at baseline, 4-weeks in treatment and after treatment. The waiting list control group was assessed at the corresponding time periods. After the seven-week treatment period the waiting list control group were offered a shortened flotation-REST treatment (four sessions), but no further data were collected. Follow-up data were collected 6-months after the end of treatment from the participants in the treatment group. The last follow-up data was received January 2015. Estimates of sample size were determined based on the results of previous studies of flotation-REST [(e. g.) [21, 23]]. No changes to the method were done after trial commencement.

### Measures

#### PSWQ—penn state worry questionnaire

The 16-item PSWQ is the most widely used assessment for pathological worry [32]. The PSWQ has shown good validity and reliability in both clinical and non-clinical samples [33, 34]. The PSWQ has also shown to be able to distinguish GAD-patients from patients with other psychiatric disorders [35]. The instrument yields a total score ranging between 16 (minimum) and 80 (maximum), with a recommended cut-off score of 45+ indicating GAD [30]. The PSWQ has been translated to Swedish and validated by Breitholtz and Rondahl [36].

#### GAD-Q-IV—the generalized anxiety disorder questionnaire 4th edition

The GAD-Q-IV is a 9-item self-report measure assessing severity of generalized anxiety syndrome (GAD) as defined by the 4th edition of the Diagnostic and Statistical Manual [1]. The GAD-Q-IV was initially developed as a screening tool for GAD [37] and in the present study the measure is used as a continuous variable by summing the responses and creating a total score ranging from 0 (minimum) to 12 (maximum). Newman et al. [29] have suggested a cut-off score of 5.7+, yielding optimal ratio between sensitivity and specificity. This dimensional scoring system has demonstrated good concurrent and discriminant validity and good test-retest reliability in non-clinical samples [29]. The GAD-Q-IV has been translated into Swedish and validated by Breitholtz and Rondahl [36].

#### MADRS-S—montgomery-asberg depression rating scale

The 9-item MADRS-S is a well-established Swedish self-report assessment of depressive symptoms [38]. The MADRS-S has shown good reliability [39] and corresponds well with clinicians’ ratings [40]. The MADRS-S yields a total score ranging from 0 (minimum) and 54 (maximum). Each item relates to symptoms of depression, which is rated from 0 to 6 by respondents regarding how well it corresponds with their experience of the last three days. Cut-off values have been established at: 0–6 = no depression; 7–19 = mild depression; 20–34 = moderate depression; 34 + = severe depression [38].

#### PSQI—the Pittsburgh sleep quality index

The PSQI is a 19-item self-report measure assessing subjective sleep quality, and is widely used in both clinical and non-clinical settings [41]. PSQI have demonstrated acceptable levels of validity and reliability in both clinical [42] and non-clinical samples [43]. The instrument measures sleep disturbance during the previous month. The PSQI gives a global score ranging from 0 (minimum) to 21 (maximum) that distinguishes between “good” and “poor” sleepers. A global score of five or higher indicates poor sleep quality [44]. The PSQI has been translated to 58 languages by the authors of the scale [41] and in the present study the Swedish version was used.

#### DERS—the dysfunctional emotional regulation scale

The DERS is a 36-item self-report measure developed by Gratz and Roemer [45] to assess emotion regulation difficulties. DERS has shown good reliability and validity [45] and gives a total score ranging from 36 (minimum) to 180 (maximum). The scoring was recoded so that higher scoring indicates greater difficulties in emotion regulation. In the present study the total score is used as a general indicator of degree of emotional regulation difficulties. The instrument has been translated into Swedish by Friberg [46] and validated with similar psychometric properties as the English version.

#### MAAS—the mindful attention awareness scale

The MAAS is a 15-item self-report measure of trait mindfulness (Brown and Ryan [31], and has been the most empirically tested measure of mindfulness [47]. The MAAS assesses the level of open and receptive attention to and awareness of ongoing experience. MAAS scores can range from 1 to 6. Higher scores indicate greater mindfulness. Pilot-studies with the Swedish version of MAAS have been carried out, indicating the instruments applicability for assessing trait mindfulness after translation [48].

#### EDN—the experienced deviation from normal state scale

The EDN is a Swedish 29-item self-report measure specifically designed to be used in flotation-REST experiments, and assess degree of relaxation and deviation from normal state experienced during the flotation session [24]. The items consist of statements such as *“I could see images clearly”*, *“It felt like I was about to fall asleep”* and *“I felt a deep peace within me”*, which were graded on VAS-scales ranging from 0 to 100 on how well they corresponded to the experience during the flotation-REST session. The EDN yields a total score by averaging the scoring from the 29 items. In earlier studies on flotation-REST [20, 49] in which the EDN has been used, Cronbachs’s alpha has been measured to 0.91–0.97, suggesting good reliability. A score of 30 on EDN at the first flotation session, and a score of 40 at the subsequent flotation sessions, is viewed as an indication of typical treatment response [24], and can be compared to resting on a bed in a dark quiet room which in general gives score of 15 [50].

#### Background information

Background information was assessed using a self-constructed questionnaire which contained questions regarding age, gender, as well as questions related to the exclusion criteria’s. In addition, other forms of ongoing therapeutic treatment were assessed by questions regarding what type of medication used, dosage, as well as if the participant received psychotherapeutic treatment, and if so, what type and to what extent they were received.

#### Flotation tanks

Flotation-tanks measuring 270 cm x 150 cm x 130 cm were used. The tanks were filled with water saturated with Epsom salt (magnesium sulphate) approximately 0.3 m in depth. The water temperature was maintained at a temperature of 35° Celsius. The tanks were insulated to keep out sound and light, and earplugs were used to further minimize sensory input. The tanks were situated in quiet rooms that are locked from the inside, but can be opened from the outside by the floating laboratory staff. Shower and toilet could be accessed in the floating rooms. The participants were instructed to shower before and after the floating session. Ventilation and light could be controlled by the participants from inside the tanks. The participants were instructed to keep the lights out in the tank if possible. Also an alarm button could be pushed from inside the tank to alert the staff in the laboratory if needed. This button was not used by any of the participants.

#### Flotation-REST intervention

The intervention for the treatment group consisted of 12 sessions (á 45 min) of flotation-REST extending over seven weeks with two sessions a week. One week was treatment free (fourth week). The main reason for having a treatment free week was so that female participants could plan the timing of their flotation treatments from the incidence of each menstrual cycle. The participants had the opportunity to sit down and relax for a while before and after the flotation-sessions, and the staff in the laboratory kept conversation with the participants to a minimum.

### Procedure

Participants signed a written informed consent upon accepting participation in the study to the effect that they had received sufficient information about the study, that they were guaranteed confidentiality, could terminate their participation without giving any specific reason, and that the data collected could be scientifically analyzed and published as long as the confidentiality of the participants were guaranteed. Before signing the informed consent, the floating laboratory at Karlstad University was shown and information about the floating intervention given. Participants were also informed that they could continue with ongoing therapeutic treatments during participation in the study.

Participants then completed the self-report measures used in the study (see Measures) except for the EDN scale. After checking that the participants met the inclusion and no exclusion criteria for the study, participants were randomized to either treatment or waiting list control condition. Participants in the waiting list control group were informed that they were entitled to a shortened treatment program (four floating sessions) after they completed the post-treatment assessments, and two additional visits to the laboratory were booked four and seven weeks later for further assessments. For the participants in the treatment group the two initial floating sessions were booked for the coming week. During the floating sessions, staffs were present in the laboratory and could be alerted with an alarm button inside the floating tank. The EDN scale was administered for the treatment group after their first floating session, followed by four weeks in treatment and post-treatment assessments (after a flotation session). Four weeks in- and post-treatment the self-report measures, except background data and EDN, were filled out again by all the participants. For the treatment group this was done in adjunction to a floating session (before), and for the waiting list control group this was done at the booked visits to the laboratory. Six months after completed treatment follow-up data, consisting of all the self-report measures at post-treatment except for EDN, was collected by mail correspondence with the participants in the treatment group. Additional data were also collected and are presented elsewhere (Jonsson & Kjellgren: A phenomenological perspective on the experience of undergoing flotation-REST treatment while having generalized anxiety disorder, in preperation).

### Ethics

The protocol for this study was approved by the Ethical Board on Experimentation on Human Subjects in Uppsala, Sweden (Dnr 2013/357). The study is registered in the Australian New Zealand Clinical Trial Registry (ACTRN12613001105730). Date of registration: 09/10/2015.

### Data analysis

A two-way mixed Pillais’ MANOVA was used, where *Time* with assessments before (Baseline), four weeks in treatment (Mid), and after treatment (Post-treatment) constituted the within group factor and where *Group* (Treatment, Waiting list control) constituted the between group factor. The primary outcome was the participants’ level of GAD-symptomatology (GAD-Q-4), and the secondary outcomes were pathological worry (PSWQ), difficulties in emotional regulation (DERS), Mindfulness (MAAS), sleep difficulties (PSQI), and depression (MADRS-S). In addition, independent sample t-tests were performed comparing the groups scoring on the dependent variables at post-treatment. For the treatment group a one-way ANOVA was conducted for the scoring on EDN for the three time periods (Baseline, Mid, Post-treatment). Paired sample *t*-test was also carried out comparing participants scoring on the dependent variables at post-treatment with 6-months follow-up scoring. This was only assessed for the treatment group to assess any lasting effects of the treatment. Chi-Square goodness of fit tests was conducted, comparing the groups in regard to received psychotherapy and the use of anxiolytic, antidepressant, as well as sleep medication at baseline and post-treatment. In addition, the criteria for “clinical significant improvement” as defined by Jacobson, Follette and Revenstor [51] were used to determine the precision of the treatment effects. This implies that treated individuals must, in addition to showing a statistically reliable change, fall within the range of a normal group, at post-treatment assessment, as indicated by for an example established cut-off values for a specific self-report measure. Clinical significant change was assessed for PSWQ, GAD-Q-IV, MADRS-S and PSQI. See Measures for the cut-off values used for these assessments.

## Results

The analysis yielded significant main effects for *Time* [*F*_(12, 33)_ = 5.69, *p* < 0.001, η_p_^2^ = .67] and *Group* [*F*_(6, 39)_ = 2.44, *p* = .042, η_p_^2^ = .27]. There was also a significant *Time x Group* interaction effect [F_(12, 33)_ = 3,87, *p* = .001, η_p_^2^ = .58). The results from the univariate F-tests are presented below. For means and standard deviations, see Table 2.

**Table 2:** Means (standard deviations) for treatment outcomes divided by time and group

	Treatment group (*n =* 24)			Control group (*n =* 22)		
Measure	Baseline	Mid	After	Baseline	Mid	After
MADRS-S	24.04 (7.46)	13.38 (5.80)	10.25 (7.56)	20.77 (7.46)	21.09 (6.67)	19.09 (7.98)
PSWQ	61.88 (11.21)	56.21 (13.93)	52.67 (12.25)	57.95 (10.29)	56.91 (10.54)	54.68 (11.47)
GAD-Q-IV	10.01 (2.20)	7.75 (3.88)	7.07 (3.02)	9.92 (2.24)	9.07 (2.88)	9.22 (3.38)
PSQI	10.58 (3.92)	6.88 (4.05)	5.71 (3.32)	9.73 (3.49)	9.18 (3.78)	8.68 (4.08)
DERS	99.67 (20.18)	85.63 (22.55)	83.04 (22.86)	97.64 (20.33)	98.86 (21.88)	97.64 (21.43)
MAAS	2.98 (0.65)	3.37 (0.79)	3.64 (1.06)	3.64 (1.06)	3.45 (0.72)	3.35 (0.81)
EDN	31.59 (15.43)	45.48 (15.43)	44.05 (16.25)			

Note. *PSWQ* Penn State Worry Questionnaire, *GAD-Q-IV* Dimensional scoring from the Generalized Anxiety Disorder Questionnaire, *MADRS-S* Montgomery-Asberg Depression Rating Scale, *PSQI* Pittsburgh Sleep Quality Index, *DERS* Dysfunctional Emotional Regulation Scale – Total mean score, *MAAS* Mindful Attention and Awareness Scale, EDN = Experienced deviation from normal state scale; Baseline = Before treatment; Mid = 4 weeks in treatment; After = Post-treatment

### Primary outcome

#### GAD-symptomatology

The analysis indicated significant difference for *Time* [*F*_(2,88)_ = 8.79, p < .001, η_p_^2^ = .167], and the descriptive data showed that GAD-symptomatology decreased. There was also a significant *Time x Group* interaction effect [*F*_(2,88)_ = 2.93, *p* < .001, η_p_^2^ = .062], and further analyses (paired-samples t-tests, 5 % level) showed that the GAD-symptomatology was significantly reduced for the treatment group (*t*_*(*23)_ = 4.47, *p* < .001), but not for the waiting list control group (t_(21)_ = 0.98, *p* > .05), when comparing baseline to post-treatment scoring. Subsequent pairwise comparisons (5 % level) of *Time* for both groups also indicated a significant decrease for the treatment group when comparing baseline to mid scoring (*p* < .05). No other comparisons for *Time* were significant (*ps* > .05).

**Fig. 2:**
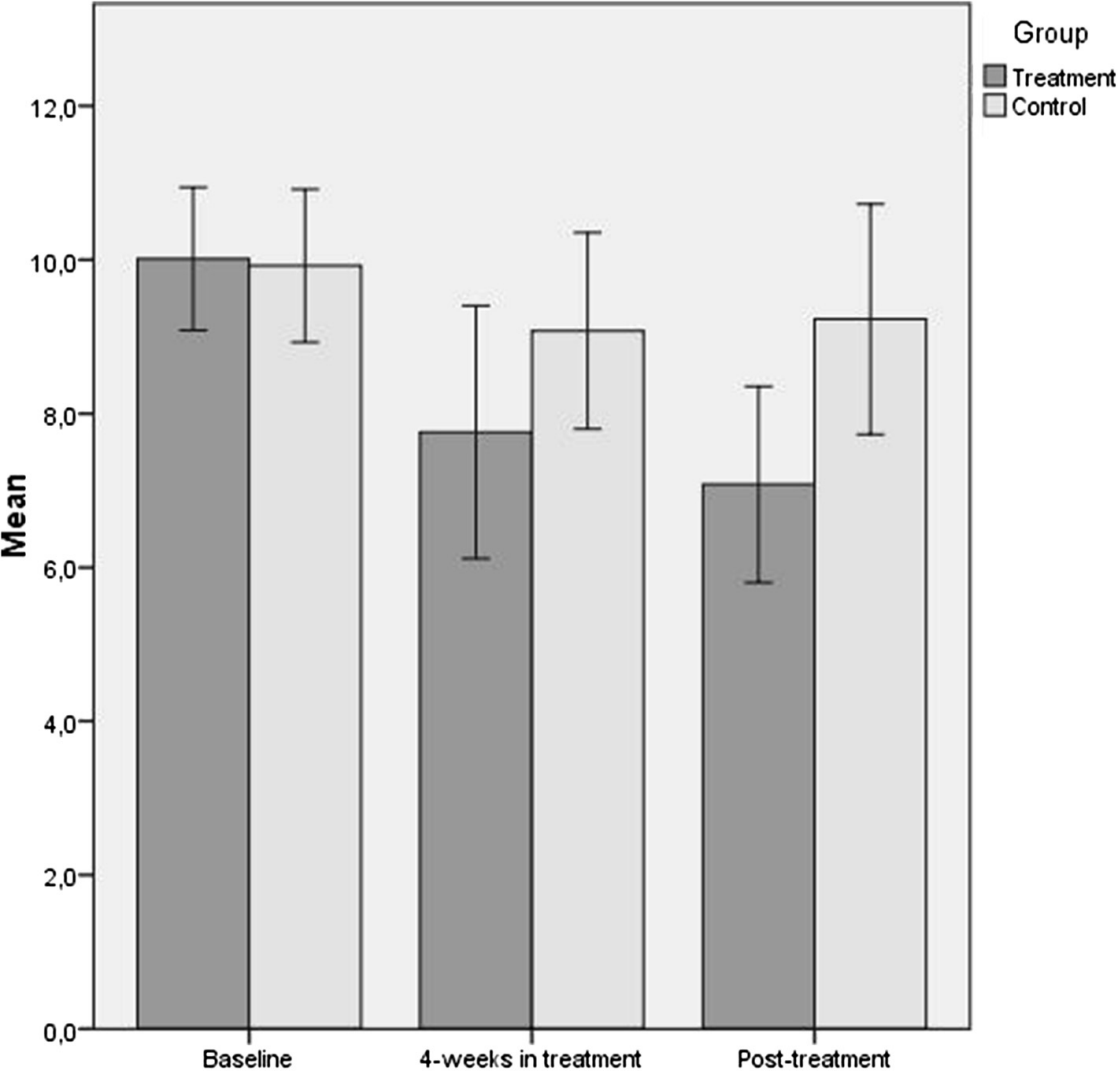
Bar-chart illustrating means for the scoring on GAD-Q-IV, with associated 95 % confidence interval, for treatment and control group at baseline, 4 weeks in treatment, and post-treatment

Comparing the groups at post-treatment (independent sample *t*-test, 5 % level) showed that the the treatment group had significantly lower GAD- symptomatology then the waiting list control group (*t*_*(44)*_ = − 2.27, *p* < 0.05). Regarding clinical significant change, nine participants (37 %) in the treatment group, and three participants (14 %) in the waiting list control group, scored below the suggest cut-off value indicating full remission of GAD at post-treatment. Figure 2 below illustrate changes in GAD-symptomatology for the two groups over time.

### Pathological worry

The analysis indicated significant difference for *Time* [*F*_(2,88)_ = 16.28, *p* < .001, η_p_^2^ = .27], and the descriptive data showed that pathological worry decreased. There was also a significant *Time x Group* interaction effect [*F*_(2,88)_ = 4.05, *p* < 0.05, η_p_^2^ = .084], and further analyses (paired-samples t-tests, 5 % level) showed that pathological worry was significantly reduced for both the treatment group (*t*_(23)_ = 5.77, *p* < .001), and for the waiting list control group (*t*_*(21)*_ = 2.15, *p <* .05), when comparing baseline to post-treatment scoring. Subsequent pairwise comparisons (5 % level) of *Time* for both groups also indicated a significant decrease for the treatment group when comparing baseline to mid scoring, and when comparing mid to post-treatment scoring (*ps* < .05). No other comparisons for *Time* were significant (*ps* > .05).

Comparing the groups at post-treatment (independent sample *t*-test, 5 % level) showed that the groups did not significantly differ (*t*_*(44)*_ = − 2.27, *p* < .05). Regarding clinical significant change, three participants (12 %) in the treatment group, and four participants (18 %) in the waiting list control group, scored below the suggest cut-off value indicating full remission of GAD at post-treatment.

### Difficulties in emotional regulation

The analyses indicated significant difference for *Time* [*F*_(2,88)_ = 7.73, *p* < .001, η_p_^2^ = .15], and the descriptive data showed that difficulties in emotional regulation decreased. The analyses also yielded a significant *Time x Group* interaction effect [*F*_(2,88)_ = 8.61, *p* < .001, η_p_^2^ = .16], and further analyses (paired-samples t-tests, 5 % level) showed that difficulties in emotional regulation was significantly reduced for the treatment group (*t*_(23)_ = 4.01, *p* < .01), but not for the waiting list control group (*t*_*(21)*_ = 0.00, p > .05), when comparing baseline to post-treatment scoring. Subsequent pairwise comparisons (5 % level) of *Time* for both groups also indicated a significant decrease for the treatment group when comparing baseline to mid scoring, (*p* < .05). No other comparisons for *Time* were significant (*ps* > .05).

Comparing the groups scoring at post-treatment (independent sample *t*-test, 5 % level) showed that the treatment group had significantly lesser difficulties in emotional regulation than the waiting list control group (*t*_*(44*)_ = − 2.22, p < .05).

### Mindfulness

There was no significant effect for *Time* [*F*_(2,88)_ = 1.39, *p* > .05, η_p_^2^ = .031], but there was significant *Time x Group* interaction effect [*F*_(2,88)_ = 9.28, *p* < .001, η_p_^2^ = .174]. Further analyses (paired-samples t-tests, 5 % level) showed that mindfulness significantly increased for the treatment group (*t*_(23)_ = − 3.00, *p* < .01), and significantly decreased for the control group (*t*_*(21)*_ = 2.36, *p <* .05), when comparing baseline to post-treatment scoring. Subsequent pairwise comparisons (5 % level) of *Time* for both groups*,* indicated a significant increase for the treatment group when comparing baseline to mid scoring (*p* < .05). No other comparisons for *Time* were significant (*ps* > .05).

Comparing the groups scoring at post-treatment (independent sample *t*-test, 5 % level) showed that the groups did not significantly differ (*t*_*(44*)_ = 1.01, p > .05).

### Sleep difficulties

The analysis indicated significant difference for *Time* [*F*_(2,88)_ = 19.52, *p* < .001, η_p_^2^ = .30], and the descriptive data showed that sleep difficulties decreased. There was also a significant *Time x Group* interaction effect [*F*_(2,88)_ = 8.76, *p* < .001, η_p_^2^ = .16], and further analyses (paired-samples t-tests, 5 % level) showed that sleep difficulties was significantly reduced for the treatment group (*t*_(23)_ = 5.87, *p* < .001), but not for the control group (*t*_*(21)*_ = 2.13, p > .05), when comparing baseline to post-treatment scoring. Additional pairwise comparisons (5 % level) of *Time* for both groups, indicated a significant decrease for the treatment group when comparing baseline to mid scoring (*p* < .05). No other comparisons for *Time* were significant (*ps* > .05).

Comparing the groups at post-treatment (independent sample *t*-test, 5 % level) showed that the treatment group had significantly lesser sleep difficulties than the waiting list control group (*t*_*(44)*_ = − 2.71, *p* < .01). Regarding clinical significant change, 13 participants (43 %) in the treatment group, and six participants (27 %) in the waiting list control group, scored below the suggest cut-off value indicating a *“good sleeper”* at post-treatment.

### Depression

The analysis indicated significant difference for *Time* [*F*_(2,88)_ = 31.60, *p* < 0.001, η_p_^2^ = .32], and the descriptive data showed that the level of depression decreased. The analysis also yielded a significant *Time x Group* interaction effect [*F*_(2,88)_ = 22.77, *p* < 0.001, η_p_^2^ = .34], and further analyses (paired-samples t-tests, 5 % level) showed that level of depression was significantly reduced for the treatment group (*t*_(23)_ = 7.24, *p* < .001), but not for the waiting list control group (*t*_*(21)*_ = 1.12, p > .05), when comparing baseline to post-treatment scoring. Subsequent pairwise comparisons (5 % level) of *Time* also indicated a significant decrease for the treatment group when comparing baseline to mid scoring, and when comparing mid to post-treatment scoring (*ps* < .05). No other comparisons of *Time* were significant (*ps* > .05).

When comparing the groups at post-treatment (independent sample *t*-test, 5 % level), it was found that the treatment group had significant lesser depression that the waiting list control group (*t*_*(44)*_ = − 3.85, *p* < .001). Regarding clinical significant change, 10 participants (42 %) in the treatment group, and two participants (9 %) in the waiting list control group, scored below the suggest cut-off value indicating a full remission of depression at post-treatment.

### Experienced deviations from normal state

Assessment of deviations from normal state during the flotation sessions (for the treatment group) indicated a main effect for *Time* [*F*_(2,22)_ = 6.57, *p <* .05, η_p_^2^ = .37], with a significant increase in EDN-scoring from baseline to mid scoring and from baseline to post-treatment scoring (*ps* < .05). There was no significant effect when comparing mid to post-treatment scoring (*p* > .05), indicating that most changes on this variable occurred in the first four weeks of treatment and then was maintained at this level for the rest of treatment (see Table 2).

### Follow-up data

In total nineteen (*n =* 19) from the treatment group returned the follow-up assessments by mail. Results indicated no significant difference (*ps* > .05) for GAD- symptomatology, pathological worry, sleep difficulties, emotion regulation difficulties or mindfulness when comparing post-treatment to follow-up scorings (See Table 3), indicating that the effects from treatment were maintained at 6-month follow-up. For depression, the scoring was higher at follow-up compared to post-treatment, and this difference was significant (*t*_*(18*_*)* = −2.90, *p* < .05), indicating that treatments effect on depression was not maintained at 6-month follow-up.

**Table 3:** Means (standard deviations) for assessed variables at post-treatment and at 6-month follow-up. Treatment group only

Measure	Post-treatment (*n =* 24)	Follow-up (*n =* 19)
GAD-Q-IV	7.07 (3.02)	6.65 (4.17)
PSWQ	52.67 (12.25)	53.06 (12.69)
DERS	83.04 (22.86)	85.12 (24.74)
MADRS-S	10.25 (7.56)	13.88 (8.10)^a^
PSQI	5.71 (3.32)	7.59 (3.95)
MAAS	3.64 (1.06)	3.61 (1.11)

Note. *GAD-Q-IV* Dimensional scoring from the Generalized Anxiety Disorder Questionnaire, *PSWQ* Penn State Worry Questionnaire, *DERS* Dysfunctional Emotional Regulation Scale – Total mean score; *MADRS-S* Montgomery-Asberg Depression Rating Scale, *PSQI* Pittsburgh Sleep Quality Index, *MAAS* Mindful Attention and Awareness Scale

^a^Significant at the 0.05 level

### Medication and psychotherapy

Chi-square goodness of fit test (5 %) indicated no significant differences when comparing the groups in regard to received psychotherapy and the use of anxiolytic, antidepressant, as well as sleep medication at baseline and post-treatment (see Table 4). One participant in the control group, but none in the treatment group, increased the dosage of anxiolytic medication during the course of the study. In addition, two participants in the treatment group decreased their dosage of sleep medication. Psychotherapy consisted of CBT,except for one participant in the control group and two participants in the treatment group that received unspecified counseling..Participants who had ongoing psychotherapy during the study met with their therapist one time a week.

**Table 4:** Percentage of participants in each group that received medication and psychotherapy

Variable	Control		Treatment		
	Baseline (*n =* 25)	Post (*n =* 22)	Baseline (*n =* 25)	Post (*n =* 24)	follow-up (*n =* 19)
Psychotherapy	36 %	36 %	28 %	20 %	16 %
Anxiolytics	28 %	22 %	20 %	16 %	16 %
Antidepressants	28 %	22 %	24 %	20 %	10 %
Sleep medication	8 %	9 %	12 %	0 %	5 %

## Discussion

The main goal of the present study was to do an initial evaluation of flotation-REST as a treatment of GAD. The sample used was screened with self-report measures extensively used to detect GAD in both clinical and non-clinical populations [29, 30]. The main findings were that flotation-REST significantly reduced both the general GAD-symptomatology, as well as several symptoms associated with the disorder, such as difficulties in emotional regulation, sleep difficulties and depression, while having ambiguous or non-existent effect on level of pathological worry and mindfulness. In addition, all improved outcome variables at post-treatment, except for depression, were maintained at 6-month follow-up assessments.

Considering that GAD has been considered a treatment resistant disorder [16, 52] it is interesting that flotation-REST significantly improved GAD-symptomatology (*η*_*p*_^*2*^ = .062), in which 37 % in the treatment group reached full remission at post-treatment (waiting list control: 14 %). Results also showed that some factors known to play an important part in maintaining GAD (pathological worry and low mindfulness) were not affected by the treatment. A possible interpretation of these results might be that treatment mainly affects physiological factors in the GAD-symptomatology, such as restlessness, fatigue and muscle tensions, thus explaining the reduction of the general GAD-symptomatology, while the core characteristic of GAD, the pathological worry, is left marginally affected. This interpretation is also supported by earlier research on flotation-REST which has shown that the most robust effects from flotation-REST are relaxation [18] and reduction of muscle tension pains [(e. g.) [24]]. Taken together this implies that additional treatment is needed to reach full remission of GAD-symptomatology by floating.

However, there was a convincing beneficial effect of flotation treatment on difficulties in emotional regulation (*η*_*p*_^*2*^ = .16), indicating that the awareness of emotions increased, and that the treatment enabled individuals with GAD to better understand and regulate their emotional responses. This is an important finding considering earlier laboratory studies, reporting that individuals who met the criteria for GAD by self-reports measures had higher levels of intensity of emotional experience than control individuals, and in addition exhibited marked difficulties in their capacity to identify, describe, and accept emotional experience [53]. Emotion regulation training has been proposed to be a potentially valuable addition to existing treatment protocols for GAD [54], in the light of this, flotation-REST might be an asset in the treatment of GAD, targeting this aspect of difficulties associated with the disorder.

The results also indicated that the flotation treatment had a strong beneficial effect (*η*_*p*_^*2*^ = .16) on sleep difficulties, in which 43 % of the participants in the treatment condition were *“good sleepers”* at post-treatment (waiting list control: 27 %). Co-existence of sleep difficulties and anxiety has been extensively reported in previous studies [(e. g.) [55]], and approximately half of individuals diagnosed with GAD report having difficulties with sleeping [56]. This underlines the importance of this finding, and suggests that individuals with GAD might benefit from flotation-REST treatment by improving their sleep.

The improvement of sleep could also have contributed to other beneficial effects, such as the lowering of depression, which was the strongest effect from treatment (*η*_*p*_^*2*^ = .34), and where 42 % of the participants in the treatment group reached full remission at post-treatment (waiting list control: 9 %), especially when considering earlier studies which have suggested that sleep difficulties could trigger depression and other forms of psychiatric diseases (e. g. [57]). The reduction of depression is also in line with earlier research on flotation-REST which has repeatedly demonstrated this effect for various populations suffering from stress related disorders [(e. g.) [20, 21]]. The observed improvements of depression at post-treatment were not maintained at 6-months follow-up, which might indicate that additional booster session is necessary to make this effect of treatment persist over time.

Regarding treatments effect on mindfulness, the groups did not significantly differ at post-treatment, despite that results indicated a considerable significant *Time x Group* interaction (*η*_*p*_^*2*^ = .17). The treatment group significantly increased their level of mindfulness when comparing baseline to post-treatment scoring, but since the groups differed significantly on this variable at baseline it is hard to draw any firm conclusion about the treatments effectiveness on this dimension.

The several positive effects of treatment observed in the present study suggest that individuals with GAD, in line with what has been confirmed in other flotation-REST studies on various patient-groups [(e. g.) [19, 23, 24]], experienced the treatment as beneficial. Possible the deep relaxation that is induced during the flotation sessions could be a contributing factor to these positive effects from treatment. This is supported by the scoring on the EDN-scale, which has been used extensively in earlier flotation-REST studies [(e. g.) [19, 20]] as an indirect measure of experienced relaxation during the flotation sessions. The significant increase over time for the treatment group on this variable, indicated a normal treatment response of flotation-REST [(e. g.) [24]].

This study is not without limitations. Even though the sample was identified with self-report measures that has been extensively used to identify GAD in both clinical and non-clinical populations, more research with clinical populations is needed to confirm that the findings is generalizable to GAD patients. The cut-off score used for the PSWQ could also be regarded as relatively low compared to some studies [(e. g.) [58]], which further underlines the importance to conduct studies with clinical populations to be able to draw any firm conclusions regarding the effectiveness of flotation-REST as a treatment of GAD. There was also a small but significant difference when comparing the groups scoring of mindfulness at baseline, although the level is to be considered low for both groups when considering normative data [31]. This group difference is still somewhat problematic, especially since mindfulness is associated with depression and pathological worry [(e. g.) [59]]. On the other hand, the scoring on the other assessments at baseline showed substantial psychological suffering for both groups, including the dimensions of pathological worry and depression, indicating that the difference on the mindfulness dimension at baseline did not have a significant impact on the other dependent variables. Furthermore, a sizeable proportion of the the participants received uncontrolled medication and/or psychotherapy. In addition, the CBT interventions received could possible differ in quality, both in regard of the competence of the therapist, and in regard of potential differences in what CBT techniques that were emphasized during these treatments. Although it can not be ruled out that the groups differed in regard to how other forms of received treatment impacted the dependent variables, the effects from these confounding variables should have been addressed by the applied randomization to the sample. That randomization worked out efficiently is also indicated by the chi-square test at baseline and post-treatment, which showed that the groups did not significantly differ in regard to received psychotherapeutic and psychopharmacological treatments. In addition, although no statistical difference was found in regard to received psychopharmacological treatment, the use of medications was somewhat higher for the control group at baseline.

Despite these limitations, the present study provides some initial data which could guide further research in the field. Considering these results, as well as that no negative side effects has been associated with the method either in present or other studies, further research should try to establish that the current findings is applicable to patients with GAD, by conducting randomized clinical trials with active control condition, and by further enhance the study design by for an example blinding the study personnel. It could also be of interest to study flotation-REST combined with a psychotherapeutic intervention, which in earlier pilot-studies [(e. g.) [27, 28] has been reported to be a combination which makes the treatment process more effective. Furthermore, flotation-REST should be explored as a complementary intervention of other types of mood and anxiety disorders, especially those in which emotional regulation difficulties, sleep difficulties and depression are central issues.

## Conclusions

The present study is the first to evaluate flotation-REST primarily as an intervention for GAD.

The findings suggest that flotation-REST has potential as a complementary treatment modality alongside existing treatment protocols for GAD, and that more studies exploring this novel approach could be a fruitful endeavor for advancing existing treatments of GAD. Considering the results, further research should focus on how the treatment could best be implemented as an intervention for improving sleep, mood and emotion regulation difficulties in individuals with GAD.
